# Key factors for sustainable working conditions in emergency departments: an EUSEM-initiated, Europe-wide consensus survey

**DOI:** 10.1097/MEJ.0000000000001159

**Published:** 2024-07-19

**Authors:** Matthias Weigl, Michael Lifschitz, Christoph Dodt

**Affiliations:** a Institute for Patient Safety, University Hospital Bonn, Bonn, Germany; b Institute and Clinic for Occupational, Social and Environmental Medicine, LMU University Hospital, LMU Munich, Munich, Germany; c Acute and Emergency Care Clinic; München Klinik Bogenhausen, Munich, Germany

**Keywords:** burnout, consensus, Delphi, emergency department, Europe, patient care, stress, survey, well-being, work-life

## Abstract

**Background and importance:**

Modern emergency medicine (EM) is a complex, demanding, and occasionally stressful field of work. Working conditions, provider well-being, and associated health and performance outcomes are key factors influencing the establishment of a sustainable emergency department (ED) working environment.

**Objectives:**

This multinational European Delphi survey aimed to identify unequivocal major factors for good and poor ED working conditions and their possible effects on health care provider well-being.

**Design/setting and participants:**

A total of 18 experts from six European countries (Belgium, Finland, Germany, Italy, Romania, and the UK) covering three different hospital sizes (small, medium, and large) in their respective countries participated in the two-round Delphi survey. All panelists held leadership roles in EM.

**Outcome measures and analysis:**

The first step involved conducting an extensive literature search on ED working conditions. The second step involved the first Delphi round, which consisted of structured interviews with the panelists. The survey was designed to obtain information concerning important working conditions, comments regarding work-life factors identified from the literature, and ratings of their importance. Interviews were transcribed and analyzed following a standardized protocol. In the second Delphi round, experts rated the relevance of items consolidated from the first Delphi round (classified into ED work system factors, provider health outcomes, and ED work-life intervention approaches).

**Results:**

A nearly unequivocal consensus was obtained in four ED work condition categories, including positive (e.g. job challenges, personal motivation, and case complexities) and negative (e.g. overcrowding, workflow interruptions/multitasking, medical errors) ED work conditions. The highly relevant adverse personal health events identified included physical fatigue, exhaustion, and burnout. Concerning intervention practices, the panelists offered a wide spectrum of opportunities with less consensus.

**Conclusion:**

Work system conditions exert positive and negative effects on the work life of ED providers across Europe. Although most European countries have varying health care systems, the expert-based survey results presented herein strongly suggest that improvement strategies should focus on system-related external stressors common in various countries. Our findings lay the scientific groundwork for future intervention studies at the local and systemic levels to improve ED provider work life.

## Introduction

Emergency medicine (EM) is an integral part of every healthcare system. Throughout Europe, increasing demands for EM have emerged due to demographic changes and the overloaded primary care sector. Such circumstances have resulted in increased numbers of elderly and multi-morbid patients and increasing rates of low-acuity visits [1–4].

Emergency departments (ED) are highly dynamic and stressful environments with specific organization- and patient-related demands for providers, which subsequently affect the health and performance of ED providers [5–8]. ED care includes exposure to various patient-related and occupational stressors, such as child sexual abuse, workplace violence, and high workloads [9–12]. Beyond the inherent challenges of ED care (e.g. time pressure or limited predictability), other work-related stressors have also been associated with poor organizational or system design (i.e. limited autonomy, social conflicts, understaffing, and communication problems) [7,13,14]. The quality of EM critically depends on stringent, timely processes. Poor EM processes contribute to adverse outcomes, such as in-hospital mortality [15] or adverse events [16].

Given the pertinent strains in ED care, work system factors that potentially place providers and patients at risk are of particular interest [1]. However, systematic investigations into ED work life and its effects on outcomes have been limited [7]. Moreover, the majority of the available studies on ED work life have been conducted outside of Europe, with only a few comparing ED work conditions among countries [14,17]. A multinational comparison, however, might be particularly helpful in identifying system-dependent effects on ED working conditions. However, international surveys that identify factors influencing general ED work life across various healthcare systems are scarce [12,14,17,18]. The current study therefore sought to identify key ED work system characteristics that might affect ED provider well-being across selected European countries.

In addition, the current study aimed to analyze how structures, processes, and outcomes in EDs are interrelated, drawing upon a resilience and sociotechnical understanding of healthcare work [19–22]. System-based approaches account for the complexities of healthcare and seek to comprehensively capture multiple factors influencing the tasks, technologies, persons, environment, and organization that constitute ED work life. Moreover, adaptive responses to expected and unexpected demands have been labeled as ‘resilience strategies’, with respective performance and operation adjustments being classified as ‘matching, extending, sustaining, or transforming’ [23]. Therefore, the interplay between four key ED socio-technological system factors, namely staff, supplies, space, and sequence (four S’s), have been considered crucial for successful adjustments in ED performance [23]. However, concurrent factors influencing ED providers’ work situation, specific staffing requirements, patient acuity, and rest-of-hospital processes have been rarely assessed [24].

To address work-related problems and develop effective interventions, best practices and successful approaches that improve ED working conditions need to be determined [22,25]. Currently, knowledge concerning specific strategies that foster ED work environments and the provision of effective support strategies have been limited [9,26]. Identification of strategies, leverage points, and skills is necessary to safeguard resilient and reliable ED care despite the ubiquitous and persistent constraints of work environments, procedures, and resources [23,27,28]. Investigations on provider work conditions can provide useful information for policy and practice recommendations, which could improve the quality of ED workplaces [25,28,29].

### Objectives and research questions

We aimed to gather data through a systematic Delphi survey on ED work life across several European countries included in the European Society for Emergency Medicine’s (EUSEM) network, with the following three research questions (RQs):

RQ1: Which ED work system conditions affect ED physicians’ well-being on the job and self-perceived quality and safety of patient care?

RQ2: Which intervention approaches are regarded as effective in improving ED physicians’ work life, well-being on the job, and self-perceived quality and safety of patient care?

RQ3: Which aspects are responsible for the success or failure of interventions aiming to improve ED working conditions and provider well-being (barriers and facilitators)?

## Methods

### Design, methods, and ethical approval

We established an exploratory, consecutive multi-step procedure that started with a literature search and was followed by a two-round mixed-method Delphi survey. We surveyed senior ED physicians as subject matter experts (SME) to determine the ED work life in their respective countries through the board of their National Society of EM. This study was conducted in accordance with the Consolidated Criteria For Reporting Qualitative Studies guidelines [30].

Ethical approval was granted by the Ethics Board of Medical Faculty, Ludwig-Maximilians-University Munich (No. 19-729). After receiving information on the study objectives and data privacy, the panelists were requested to sign the informed consent form. Answers were collected anonymously. Participants received no financial compensation.

### Procedure and data acquisition

The first step involved conducting a literature search for relevant studies on ED working conditions and respective interventions for their improvement (in literature databases *PubMed* and *Web of Science*). This step aimed to establish an extensive unsystematic overview on the spectrum of previously reported ED work-life factors. We collected peer-reviewed publications on ED work stress, provider well-being, and associated performance outcomes and compiled a list of aspects and constructs indicative of each ED work-life area of interest. This list (for an extended list see, Supplementary Appendix A-2, Supplemental digital content 1, http://links.lww.com/EJEM/A445; for the search terms, see Supplementary Appendix A-3, Supplemental digital content 2, http://links.lww.com/EJEM/A446) was the basis for a preliminary interview guideline, which was tested through pilot interviews involving three ED senior physicians. We tested the guideline for feasibility and comprehensiveness and requested all three interviewees for feedback concerning the clarity of the questions, potential misunderstandings, and perceived lack of content. Statements were transcribed verbatim and analyzed for feedback on improvement needs. We then made some adjustments to enhance the clarity and presentation of the questions.

Subsequently, a Delphi-consensus process that consisted of two rounds was launched. A purposive snowball sampling approach was used to identify panelists from different EUSEM national society member states. Panelists were selected by one of the study initiators (author C.D.) who serves as a board member of the EUSEM and contacted national representatives of the EUSEM council. Through personal information and internal mailings, study information was distributed among EUSEM-affiliated ED professionals. The only criterion was holding a leadership position in an ED within their respective country. We then collected positive responses, after which a convenience sample was recruited. Eventually, we established a balanced, Europe-wide distribution of nations from the South East (Romania), South (Italy), Central (Belgium, Germany), West (UK), and North of Europe (Finland). Moreover, we sought to balance the number of panelists from large (>600 beds), middle-sized (200–600 beds), and small hospitals (<200 beds) for each country. To this end, three interviewees were selected for each country.

In the first Delphi round, video-based and semi-structured interviews were conducted individually using the interview guidelines. Participants were then interviewed via semi-open questions and were also asked to rate their opinion regarding certain aspects of ED work life on a Likert scale. Verbal responses were recorded and transcribed using content-related and semantic transcription rules [31]. Responses were interpreted by one study team member (author M.L.). To ensure consistency and reliability, the first three transcripts were coded together by a second experienced coder (author M.W.). Conditions ranked highly and topics mentioned most frequently were taken to round 2.

In the second Delphi round, conditions and influence factors reported to be important for provider working conditions in the first round were then presented again to all panelists. The relevance of each item was evaluated by rating them on a 5-point Likert scale from 1 (highly irrelevant) to 5 (highly relevant) (instruction: ‘Please rate the relevance for each of the following items’). Data were acquired between January 21 and August 22. Second-round responses were gathered individually via an online survey platform (Unipark, Tivian Inc.).

### Material and measures

First-round interviews aimed to cover key factors for ED providers’ work life, well-being, and associated patient care outcomes. The following content areas were captured:

(Area 1) General provider- and patient-related work-life conditions in ED work (including positive and negative aspects) and adverse provider health outcomes (i.e. physical, mental, psychosomatic, and behavioral health),

(Area 2) Improvement and intervention practices at the organization, team, and individual levels to improve ED physicians’ work life, well-being, and self-perceived quality and safety of patient care,

(Area 3) Perceptions of aspects for success and failure, including contextual influences at the macro-, meso-, and micro-system levels in EDs.

In the first round, we asked open questions for each domain. After obtaining open answers, we provided further examples from our preestablished list (based on our literature review, see Supplementary electronic Appendix A-1, Supplemental digital content 3, http://links.lww.com/EJEM/A447) to facilitate further exploration. We further assessed participants’ experiences and appraisal of COVID-19 pandemic-related challenges in ED work. For brevity, this information was not analyzed and reported here. In the second round, items were rated according to relevance.

Additionally, the following sociodemographic and contextual information was obtained from the participants: (1) academic degree, current professional role in the ED, and (2) characterization of hospital and ED (i.e. size and number of patients). In both rounds, the panelists had the option to provide further comments (via verbal statements in round 1 and free-text fields in round 2).

### Participants

Based on the recommendation of the EUSEM council members from the respective European countries, we recruited three ED physicians from basic, general, and maximum care level EDs. The following inclusion criteria were applied: (1) senior physicians actively holding a leadership role in hospital-based emergency care (i.e. either in a consultant, assistant medical director, or head of department), (2) profound experience with and knowledge of hospital-based EM, and (3) sufficient English language skills (i.e. sufficient oral and written English skills for interview purposes).

### Analyses

In round 1, written interview data were analyzed using qualitative content analyses (i.e. deductive and inductive thematic approaches), with statements being grouped and consolidated as main conditions and influence factors [32]. Two members of the study team (M.L. and M.W.) coded the answers, consolidated the main themes, and drafted a preliminary set of conditions for round 2 surveys. In round 2, panelists’ relevance ratings were analyzed using the following metrics: means, standard deviations, and consensus [defined a priori as 80% of the panelists responding with 4 (relevant) or 5 (highly relevant)] [33].

## Results

### Data and participant characteristics

The first Delphi round accumulated a total of 1534 min of interview material, with each interview lasting between 61 and 120 min (mean = 90.2). For the second round, each interview lasted between 5.7 and 34.8 min (mean = 15.7).

**Fig. 1:**
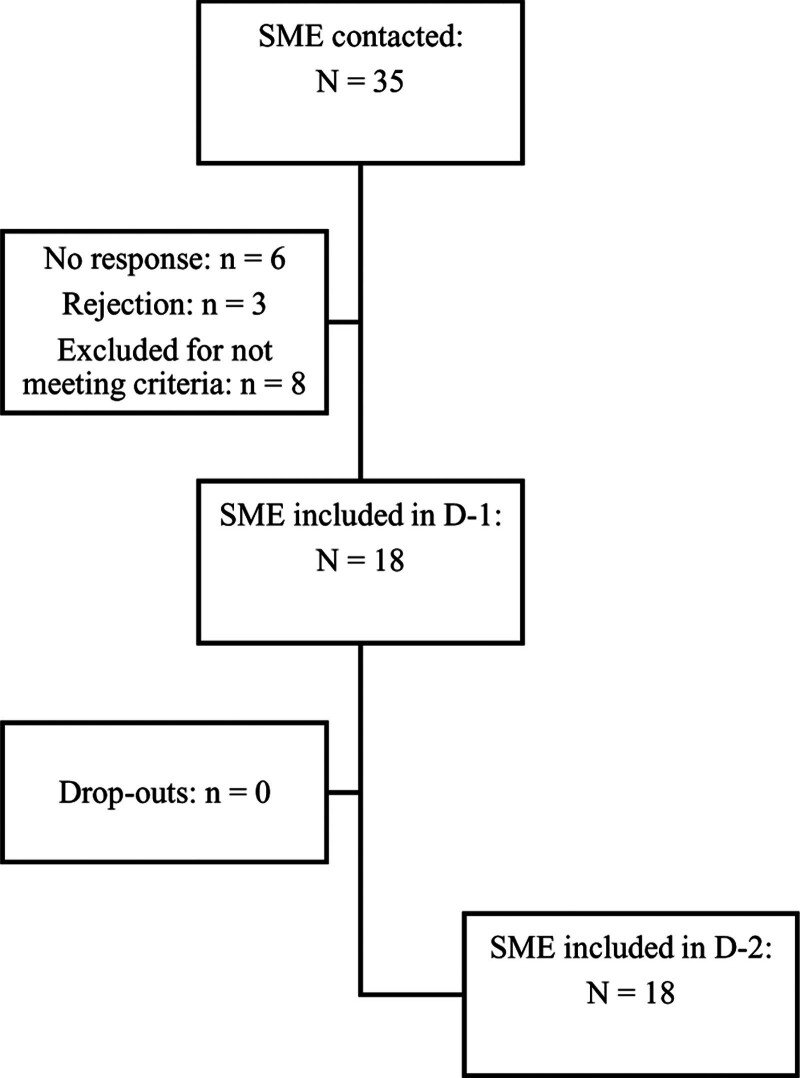
Overview of the recruitment procedure and sample description. SME, subject matter experts; D-1/-2, Delphi round one/two.

SME included 18 senior EM physicians from the following six European countries (Fig. 1): Belgium, Finland, Germany, Italy, Romania, and the UK (with each country having three panelists). From the initial potential interviewee candidate pool, six did not reply to our inquiry, three rejected it, and eight were excluded for not meeting at least one criterion (i.e. still undergoing specialty training, no leadership role in the ED, expert already available for their country, and ED size).

Among the included panelists, 11 (61.1%) were ED heads (i.e. directors), whereas seven (38.9%) were senior physicians (i.e. consultants). Gender distribution was five females/13 males. All panelists were currently working in the ED. Among the studied panelists, nine (50%) had an EM specialty degree, seven (38.9%) had a PhD title, seven (38.9%) had a current academic affiliation (e.g. full or adjunct professor) or was a faculty member, seven (16.7%) had an additional medical academic degree (e.g. in medical education), and one (5.6%) had an additional nonmedical academic degree. Moreover, six (33.3%) SMEs reported professional roles in professional societies, nine (50%) were involved in EM professional activities nationally, and two (11.1%) were involved in international activities related to EM. Concerning current employers, seven panelists (38.9%) worked in large-sized, maximum care (level 1) hospitals (size range: 650–2395 hospital beds); five (27.8%) worked in medium-sized, general care level facilities (size range: 300–564 beds); and five (33.3%) worked in small-sized, basic care (level 3) hospitals (size range: 200 to 337 hospital beds). Altogether, the reported medium annual patient volume ranged from 31 000 to 110 000.

### Relevant indicators of emergency department work system factors (research question 1)

The first round data for all 18 interviewees was eventually collated into 195 statements. Of these, 102 statements were transformed into survey items for the second round.

### Relevance ratings for positive and negative aspects of emergency department work

In the second round, all 18 panelists provided their evaluation on the relevance of various ED work system conditions. The following discussions present the relevance statements and consensus metrics.

Concerning the positive aspects of ED work, high ratings were obtained for ‘job challenges’ (i.e. ‘variation, interdisciplinary interaction’, mean = 4.7, 100% consensus), ‘job intellectuality’ (mean = 4.6, 94%), and ‘job control’ (mean = 4.4, 100%). Furthermore, ‘personal work ethics/motivation’ (mean = 4.4, 94%), ‘work experience, skill utilization’ (mean = 4.4, 89%), and ‘resilience’ (mean = 4.4, 89%) were mentioned (Table 1).

**Table 1:** Relevance ratings and consensus concerning ED work-life factors and outcomes

Category and factors	Metrics			
	M	95% CI(Min, Max)	SD	Consensus (%)
Positive general aspects in ED work				
Job challenge: variation and/or interdisciplinary interaction	4.7	(4.5, 4.9)	0.5	100
Job intellectuality	4.6	(4.3, 4.8)	0.6	94
Job control: participation in decision-making	4.4	(4.2, 4.7)	0.5	100
Task significance	4.3	(4.1, 4.6)	0.6	94
Job autonomy	4.0	(3.7, 4.3)	0.6	83
Positive provider-related aspects in ED work				
Personal work ethic and/or motivation	4.4	(4.1, 4.8)	0.8	94
Work experience and/or utilization of skills	4.4	(4.1, 4.7)	0.7	89
Resilience and coping strategies	4.4	(4.1, 4.7)	0.7	89
Teamwork, social climate, and extracurricular activities	4.3	(4.0, 4.5)	0.6	94
Positive coworker relationships	4.2	(3.8, 4.6)	0.9	83
Positive aspects of ED patient-provider interaction				
Patient case complexity	4.2	(3.9, 4.5)	0.6	89
Therapy efficacy: seeing patients getting better	3.9	(3.5, 4.4)	0.9	67
Grateful patient feedback	3.6	(3.1, 4.0)	1.0	67
Communication: social interaction with patients and/or families	3.6	(3.2, 4.0)	1.0	56
Negative general aspects in ED work				
Overcrowding	4.7	(4.4, 4.9)	0.6	94
Workflow interruptions and/or multitasking	4.6	(4.3, 4.8)	0.6	94
Time pressure and/or lack of breaks	4.4	(4.1, 4.7)	0.7	89
Employee turnover and understaffing	4.4	(4.0, 4.7)	0.8	83
Chronic cognitive workload	3.8	(3.3, 4.2)	0.9	67
Work-life imbalance	3.8	(3.3, 4.3)	1.1	67
Shift work	3.4	(2.9, 3.9)	1.1	50
Negative provider-related aspects in ED work				
Medical errors	4.1	(3.7, 4.5)	1.0	83
Lack of communication and feedback culture	3.9	(3.4, 4.3)	0.9	72
Task overlap and interaction deficits with other specialties	3.8	(3.4, 4.2)	0.9	72
High pressure to take far-reaching decisions	3.8	(3.3, 4.3)	1.1	67
Tense atmosphere	3.7	(3.1, 4.2)	1.1	72
Lack of resilience and coping mechanisms	3.7	(3.3, 4.1)	0.9	61
Insufficient supervision	3.6	(3.3, 3.9)	0.8	56
Negative aspects of ED patient-provider interaction				
Violence: verbal and/or physical	4.0	(3.4, 4.5)	1.1	72
Legal consequences of conflicts	3.7	(3.1, 4.2)	1.2	67
Minor complaints without the need of ED treatment	3.6	(3.1, 3.9)	0.9	67
Ungrateful feedback	3.6	(3.0, 4.1)	1.2	67
Excessive claims	3.4	(2.8, 4.1)	1.3	56

N = 18. Scale range ‘1—highly irrelevant’ to ‘5—highly relevant’.

Consensus indicates the percentage of panelists responding with 4 = ‘relevant’ or 5 = ‘highly relevant’.

CI, confidence interval; ED, emergency department; M, mean.

Concerning negative conditions, the highest relevance ratings were retrieved for ‘overcrowding’ (mean = 4.7, 94%), ‘workflow interruptions, multitasking’ (mean = 4.6, 94%), ‘time pressure’ (mean = 4.4, 89%), and ‘employee turnover, understaffing’ (mean = 4.4, 83%). Additionally, ‘medical errors’ (mean = 4.1, 83%) were highly relevant.

### Relevance ratings for adverse personal health events of emergency department work

Concerning adverse ED provider health outcomes, the highest relevance was reported for ‘exhaustion, mental fatigue’ (mean = 4.5, 94%) and ‘burnout’ (mean = 4.4, 83%). Regarding physical health, ‘physical fatigue’ was the highest (mean = 4.1, 83%). Concerning psychosomatic and behavioral health outcomes, ‘insomnia’ (mean = 4.0, 72%) and ‘intentions to leave/reduce hours’ (mean = 3.9, 72%) were the highest (Table 2).

**Table 2:** Relevance ratings and consensus concerning health outcomes of ED work

Category and factors	Metrics			
	M	95% CI (min, max)	SD	Consensus (%)
Adverse physical health outcomes of ED work				
Physical fatigue	4.1	(3.7, 4.4)	0.8	83
Musculoskeletal pain	3.2	(2.7, 3.7)	1.1	33
Injuries (e.g. needle stick, trauma, violence)	3.1	(2.6, 3.6)	1.1	33
Adverse mental health outcomes of ED work				
Exhaustion and/or mental fatigue	4.5	(4.2, 4.8)	0.6	94
Burnout	4.4	(4.1, 4.8)	0.8	83
Compassion fatigue, pessimism, and/or cynicism	3.9	(3.6, 4.3)	0.8	78
Anxiety	3.6	(3.2, 3.9)	0.8	56
Depression, sadness and/or low mood	3.3	(2.9, 3.8)	0.9	33
Adverse psychosomatic health outcomes of ED work				
Insomnia (sleep deprivation and/or disturbance)	4.0	(3.6, 4.4)	0.9	72
Cardiovascular consequences: hypertension and/or tachycardia	3.3	(2.8, 3.8)	1.1	44
Gastroesophageal reflux disease (GERD)	3.1	(2.6, 3.5)	1.0	33
Eating disorders	2.9	(2.4, 3.4)	1.0	28
Adverse behavioral health outcomes of ED work				
Intentions to leave or reduce working hours	3.9	(3.5, 4.3)	1.0	72
Dropouts/opt-outs (= quitters)	3.8	(3.5, 4.2)	0.8	78
Sick leaves	3.4	(2.9, 3.8)	1.0	61
Substance abuse	2.9	(2.4, 3.3)	1.0	28

N = 18. Scale range ‘1—highly irrelevant’ to ‘5—highly relevant’.

Consensus indicates the percentage of respondents responding with 4 = ‘relevant’ or 5 = ‘highly relevant’.

CI, confidence interval; ED, emergency department; M, mean.

### Ratings for effective intervention practices to improve emergency department work life (research question 2)

Concerning the implemented ED work-life improvement practices at the organization level, various items were identified in round 1. As depicted in Table 3, relevance ratings in round 2 were highest for ‘emergency care as autonomous specialty’ (mean = 4.4, 94%), formulation of ‘evidence-based SOPs, training concepts’ (mean = 4.3, 83%), ‘ED reorganization, modernization’ (mean = 4.1, 89%), and ‘adaptable staff, duty rostering’ (mean = 4.1, 78%). At the team level, the highest ratings were retrieved for ‘(simulation-based) skills trainings’ (mean = 4.3, 89%), ‘debriefings after critical events’ (mean = 4.2, 89%), and ‘regular feedback from mentors’ (mean = 4.1, 72%). Lower relevance was attributed to individual-level approaches but was highest for ‘private sport activities’ (mean = 3.6, 56%).

**Table 3:** Relevance ratings and consensus concerning ED work-life factors and outcomes

Category and factors	Metrics			
	M	95% CI (min, max)	SD	Consensus (%)
Improvement and intervention practices at the organization level				
Emergency care as autonomous specialty	4.4	(4.0, 4.7)	0.8	94
Evidence-based standard operating procedures (SOPs) and/or training concepts	4.3	(3.9, 4.6)	0.7	83
ED reorganization and/or modernization	4.1	(3.7, 4.4)	0.7	89
Adaptable staff and duty rostering	4.1	(3.6, 4.6)	1.0	78
Critical Incident Reporting System (CIRS)	3.9	(3.4, 4.4)	1.1	72
Out-of-ED ambulatory care and/or medical assessment units	3.8	(3.3, 4.3)	1.1	72
Limited on-call duties, night or weekend shifts, over-hours	3.7	(3.2, 4.2)	1.0	67
Quality circles and tracking of key performances (e.g. Morbidity-Mortality-Improvement conferences)	3.5	(2.8, 4.2)	1.5	56
National Emergency Medicine Society networking platforms	3.4	(2.9, 3.9)	1.1	50
Employee surveys	3.3	(2.8, 3.8)	1.0	44
Offer of mental health interventions	3.1	(2.6, 3.6)	1.0	39
Regular occupational health checks	2.8	(2.3, 3.3)	1.0	22
Improvement and intervention practices on team level				
(Simulation-based) Skills training (e.g. resuscitation, CRM)	4.3	(3.9, 4.6)	0.8	89
Debriefings after critical events with potential posttraumatic consequences	4.2	(3.8, 4.6)	0.8	89
Regular feedback from mentors	4.1	(3.6, 4.6)	1.0	72
Inter-professional educational initiatives for physicians and nursing	3.8	(3.2, 4.3)	1.2	61
Nurse practitioners (e.g. wound and/or pain care)	3.3	(2.8, 3.8)	1.2	50
Physician-assisted triage	3.2	(2.6, 3.8)	1.3	39
Improvement and intervention practices on individual level				
Private sports activities: individual and/or group-based	3.6	(2.9, 4.1)	1.3	56
Acute mental occupational health services (e.g. psychotherapy)	3.1	(2.4, 3.6)	1.3	44
Hospital-initiated mental health protection programs	3.1	(2.5, 3.6)	1.2	39
Private activities to prevent mental illness	3.1	(2.4, 3.7)	1.4	39
Acute physical occupational health services (e.g. physiotherapy)	2.8	(2.2, 3.4)	1.3	28
Hospital-initiated physical health protection programs	2.7	(2.2, 3.3)	1.3	28

N = 18. Scale range ‘1—highly irrelevant’ to ‘5—highly relevant’.

Consensus indicates the percentage of respondents responding with 4 = ‘relevant’ or 5 = ‘highly relevant’.

CI, confidence interval; ED, emergency department; M, mean.

### Barriers and facilitators for emergency department work life interventions (research question 3)

Given that the answers for the first round were considerably heterogeneous, we reported statements for key contents and clusters in round 1 (i.e. we did not deploy the statements in the second round for further evaluation).

Concerning potential success factors, a broad list was compiled, with immediate actions after incidents (stated as hot debriefings) or within a short period afterward (cold debriefings with Morbidity & Mortality conferences) being deemed effective. Moreover, inter-professional training approaches and support from hospital organizations (e.g. continuous modernization, ongoing investment of resources, and enforcement of zero-violence policies) were mentioned. Peer activities and an open culture of psychological safety that facilitates discussions regarding stress or well-being issues (e.g. in weekly meetings) were perceived as supportive.

Concerning potential barriers, various issues were mentioned, including increasing workload, organizational and logistic challenges, collaboration difficulties with non-ED specialties and functions, staff turnover (i.e. due to staff rotation), understaffing, difficulties in decision-making throughout the course of intended improvement practices, prolonged or delayed timelines for intervention projects, and poor implementation of technological advancements (e.g. electronic health records) due to the lack of staff and training.

## Discussion

Despite the abundance of literature on work conditions in EM, studies providing generic guidance concerning the most relevant ED work system factors have been considerably lacking. To overcome this shortcoming, the current collaborative study with the EUSEM network established a panel of ED physicians to consolidate the most relevant work-related factors influencing ED care. The present study provides an expert-based consensus on key ED work system factors across six European countries.

Our first aim was to identify ED work system conditions that affect ED physicians’ well-being while working. The consensus procedure revealed certain issues, most of which impact work life according to the ED experts. This adds to previous studies on stressors and challenges inherent to acute medical work settings [7,8,12,25]. Panelists’ ratings corroborate the eminent role of work conditions in EDs, which consist of positive and negative factors [6]. Among the positive factors, variability and interdisciplinary collaboration, job intellectuality, job control, and good personal work ethics and individual motivation have been mentioned [14]. This finding corroborates the results of previous studies, which show that ED work can be highly challenging and requires quick thinking, problem-solving, and decision-making skills with broad opportunities for learning and skill development [7]. Among the negative influence factors, the most relevant stressors included ED overcrowding, workflow interruptions, multitasking, time pressure, employee turnover and understaffing, and medical errors, all of which had significant potential in aggravating providers’ job dissatisfaction, fatigue, and burnout [7,12,14,17,34]. Moreover, overcrowding has been a well-known concern that can lead to delays in care, increased waiting times, and low patient satisfaction [14,15,35]. Frequent workflow interruptions and multitasking are inherent to ED work given the recurrent need to attend to multiple patients simultaneously [36]. Overall, factors reported to influence work life encompass a broad spectrum of positive and negative conditions, highlighting the various work-related challenges faced by ED professionals [5]. While the stated positive aspects of ED work life can be attributed to personality aspects, which incorporate high individual motivation, team orientation, and a decisive character, the negative effects depend on external and structural factors caused by the health care system, which can hardly be controlled by individual healthcare workers. Eventually, this may contribute to experiences of moral injury among individual ED professionals [37].

Research suggests that members of the ED staff experience high levels of job stress, which can promote physical and mental health problems [34,38,39]. Our panelists deemed psychological health outcomes, such as provider exhaustion, mental fatigue, and burnout, to be the most relevant. This finding underscores previous findings suggesting that ED professionals commonly experience fatigue and burnout [34]. Physical fatigue and sleep-related problems had also been deemed highly relevant. Concerning turnover intentions, previous investigations suggested that ED staff members reported high levels of turnover and job dissatisfaction [39,40]. Nonetheless, we also observed substantial disagreement regarding health outcomes, such as substance abuse (i.e. with 28% consensus and medium rating). Although this behavior has been frequently discussed as a serious problem and common coping method among healthcare providers (and ED physicians) in high-strain work environments, reliable prevalence data, effective monitoring, and mitigation measures have still been lacking [41,42].

Our second research question aimed to establish an ED expert-based recommendation on interventions deemed (in)effective in promoting ED work life. To this end, a heterogeneous list of improvement practices was generated. Our findings suggest that ED contexts are variable, with diverse local requirements for improvements or mitigation measures. Thus, system-oriented approaches that consider local needs are most likely to reduce everyday work stress [8,22,25]. Moreover, the thematic breadth and variance in ratings on perceived relevance suggest that a combination of various measures across different levels (i.e. individual, workplace, organization, and advocacy/policy) would be more effective than individual solutions [43,44]. High ratings were observed for organization-level improvement practices, such as regulatory measures; EM as a distinct specialty; formulation of standard operating procedures and training concepts; and efforts in ED reorganization, modernization, and adaptable staff and duty rostering [8]. However, careful consideration is warranted when interpreting our results concerning EM as an autonomous specialty given that exclusively dedicated ED physicians were surveyed, and other stakeholders (also outside of EM) may hold significantly different perspectives and viewpoints [45]. Nonetheless, EM as a distinct medical specialty was conceived as a key factor for improving working conditions. Although specialists in EM work efficiently under difficult circumstances, which helps reduce their overall workload, they are also aware of the specific need to maintain resilience individually and collectively as a team. EM specialists might be also more aware of specific educational and training needs, including simulation, debriefing, and so on, all of which have been proposed by the panelists. Additionally, recognition as an EM specialist might be perceived as a token of esteem, stimulating positive motivation for the job. Therefore, we further assumed that ED physicians may appraise EM as a distinct profession given that specialty recognition and care provided by trained EM physicians are deemed particularly valuable for efficacy, effectiveness, and value of acute care, especially considering the growing needs for EM and trauma care, as well as the perpetual demand for fast and cost-effective utilization of limited healthcare resources [46]. Team-level interventions, such as (simulation-based) skills trainings, debriefings, and regular feedback from mentors, were also deemed effective by the panelists. These efforts address everyday ED teamwork, communication, and leadership practice.

Individual-level interventions that reduce work stress were rated to be of lower relevance, which contradicts current studies on the beneficial effect of educational style or mindfulness-based interventions [26]. Nonetheless, our results suggest that professionalized approaches utilizing a broader set of improvement approaches applied either in isolation or combination, might improve working conditions specific to the EM environment [8,22].

Concerning our third research question, this survey sought to consolidate experiences on facilitators and barriers for effective ED work-life improvement practices. Our panelists’ answers were heterogeneous, resulting in a diverse set of potentially supportive or hindering influence factors during implementation. Our findings should therefore be considered preliminary and should require more in-depth investigations. Notwithstanding, collated statements allude to the complexities and multi-layered intricacies for sustainable implementation of interventions targeting ED providers’ work life. The identified factors highlight the diverse challenges associated with interventions in this domain, emphasizing the need for comprehensive, system-oriented approaches to successfully design and secure these improvements, in full partnership with all stakeholders [43,44]. Nonetheless, the scope of the reported factors proposes several facilitators and barriers that should be considered in the design of interventions [26]. Additionally, insights stemming from implementation science and organizational change are critical for the respective ED work-life interventions.

### Limitations

Although the Delphi survey allows for an expert-based consensus on key influences for healthy EM work environments in Europe, the grading of their importance is based on the subjective views of a limited number of experienced professionals who possess in-depth insights into EM care practices in their respective countries. Further intervention studies seeking to mitigate ED work stress are needed to objectify the importance of the identified working conditions [22]. Only a limited sample of key informants from the respective European countries was surveyed, thereby reducing the statistical power of the current study. Additionally, focus on senior ED providers may limit external validity. Their views may differ from junior physicians and younger trainees who may experience other stressors, such as longer shifts or higher workloads [13], mistreatment or career choice regret [38], and burnout [39]. Moreover, providers from other key professions involved in ED care were not involved. A purposive snowball sampling approach was established, potentially introducing selection bias. Moreover, the six countries included herein do not fully represent the entirety of Europe. Although this study sought to cover an extended scope of factors potentially influencing ED work life, it did not consider national or macro-level determinants on ED providers’ work life (e.g. specific regulatory or national health-system factors) [12]. A Delphi process has advantages in establishing consensus; however, this process may dilute valuable experiences of individual panelists who could provide important insights into experiences in specific European countries. Furthermore, the consolidation process consisted of two Delphi rounds with restricted panel re-confrontation compared with multi-round approaches. Studies have acknowledged the need for careful consideration of the criteria for consensus (i.e. 80%) and procedures to establish consistency in the formulation of statements (in round 1). Future studies may use alternate criteria to obtain consensus and metrics [33,47].

### Implications

Sustainable work environments for and well-being of ED providers are vital for safe and effective emergency care. Reducing the risk of preventable injuries to patients and providers requires an understanding of the underlying causes, modifying work practices and culture, and promoting staff engagement around common goals and measures [48]. Future intervention approaches need to consider the identified challenges associated with EM. The current survey shows that emergency physicians are highly motivated; however, concurrent stressors, such as crowding and multitasking, contribute to critical health ramifications, including burnout and exhaustion. These work-related factors are ubiquitous and seem to occur in different healthcare systems throughout Europe. Therefore, this EUSEM survey may help decision makers develop targeted strategies to improve working conditions in the field of EM.

### Conclusion

After establishing a consensus among ED physicians from six different European countries, we compiled a list of relevant work-life conditions, provider health outcomes, and respective intervention and improvement approaches to promote ED work-life. Various key working conditions with considerable consensus that may be utilized in future surveys on ED work life had been identified. Our findings may also help serve as an evidence base for interventions required to ensure sustainable working conditions in EDs. The empirical insights provided herein may also facilitate the development of focused measures to investigate ED work system factors across various national settings with Europe-wide commonality and relevance.

## Acknowledgements

This work has been part of the Doctoral Thesis Requirements for Michael Lifschitz (Medical Faculty, LMU Munich). Preliminary results were presented at the European Emergency Medicine Day on 27 May 2022, and at the EUSEM Congress in Berlin, Germany, on 19^th^ October 2022. We gratefully acknowledge both time and expertise of contributing panelist experts.

This work was partially funded by the Munich Center for Health Sciences (MC-Health) to M.W. and was supported by the Open Access Publication Fund of the University of Bonn.

Upon reasonable request, additional data can be obtained from the authors. This includes (1) list of preliminary responses (from Delphi round 1), as well as (2) complete list of work system items for evaluation (Delphi round 2).

### Conflicts of interest

C.D. is a member of EUSEM and involved in the EUSEM Working Group on Professional Well-being. For the remaining authors, there are no conflicts of interest.

## Supplementary Material


