# Supplementary material for: Key factors for sustainable working conditions in emergency departments: an EUSEM-initiated, Europe-wide consensus survey
Source: Eur J Emerg Med. 2024 Jul 19;32(1):29–37. doi: 10.1097/MEJ.0000000000001159 (PMC11665970; doi:10.1097/MEJ.0000000000001159)
Supplement: Supplementary file 1 [file ejem-32-29-s001.pdf]

Appendix A-2

List of references used for search of relevant literature for development of interview guideline (round 1)

1. Iqbal, S. and L. Pipon-Young, *The Delphi method.* Psychologist, 2009. **22**: p. 598-601.
2. Schneider, A. and M. Weigl, *Associations between psychosocial work factors and provider mental well-being in emergency departments: A systematic review.* PLoS One, 2018. **13**(6): p. e0197375.
3. Sakr, M. and J. Wardrope, *Casualty, accident and emergency, or emergency medicine, the evolution.* J Accid Emerg Med, 2000. **17**(5): p. 314-9.
4. Exadaktylos, A.K. and H. Zimmermann, *[Interdisciplinary emergency response units: he who comes too late is punished by life and or by the hospital management!].* Dtsch Med Wochenschr, 2009. **134**(23): p. 1236-7.
5. Carayon, P., et al., *Work system design for patient safety: the SEIPS model.* Qual Saf Health Care, 2006. **15 Suppl 1**: p. i50-8.
6. Arora, M., et al., *Review article: burnout in emergency medicine physicians.* Emerg Med Australas, 2013. **25**(6): p. 491-5.
7. Johnston, A., et al., *Review article: Staff perception of the emergency department working environment: Integrative review of the literature.* Emerg Med Australas, 2016. **28**(1): p. 7-26.
8. Weigl, M., et al., *Work stress, burnout, and perceived quality of care: a cross-sectional study among hospital pediatricians.* Eur J Pediatr, 2015. **174**(9): p. 1237-46.
9. Behringer, W. and C. Dodt, *[Physician staffing and shift work schedules : Concepts for emergency and intensive care medicine].* Med Klin Intensivmed Notfmed, 2020. **115**(6): p. 449-457.
10. Wrede, J., H. Wrede, and W. Behringer, *Emergency Department Mean Physician Time per Patient and Workload Predictors ED-MPTPP.* J Clin Med, 2020. **9**(11).
11. Weigl, M. and A. Schneider, *Associations of work characteristics, employee strain and self-perceived quality of care in Emergency Departments: A cross-sectional study.* Int Emerg Nurs, 2017. **30**: p. 20-24.
12. Boutou, A., et al., *Burnout syndrome among emergency medicine physicians: an update on its prevalence and risk factors.* Eur Rev Med Pharmacol Sci, 2019. **23**(20): p. 9058-9065.
13. Holden, R.J., et al., *SEIPS 2.0: a human factors framework for studying and improving the work of healthcare professionals and patients.* Ergonomics, 2013. **56**(11): p. 1669-86.
14. Carayon, P., et al., *Human factors systems approach to healthcare quality and patient safety.* Appl Ergon, 2014. **45**(1): p. 14-25.
15. Carayon, P., et al., *SEIPS 3.0: Human-centered design of the patient journey for patient safety.* Appl Ergon, 2020. **84**: p. 103033.

16. Weigl, M., et al., *Workflow interruptions and stress atwork: a mixed-methods study among physicians and nurses of a multidisciplinary emergency department.* BMJ Open, 2017. **7**(12): p. e019074.
17. Schneider, A., et al., *Physicians' and nurses' work time allocation and workflow interruptions in emergency departments: a comparative time-motion study across two countries.* Emerg Med J, 2020.
18. Weigl, M., et al., *Workflow disruptions and provider situation awareness in acute care: An observational study with emergency department physicians and nurses.* Appl Ergon, 2020. **88**: p. 103155.
19. Schneider, A., M. Wehler, and M. Weigl, *Provider interruptions and patient perceptions of care: an observational study in the emergency department.* BMJ Qual Saf, 2019. **28**(4): p. 296-304.
20. Weigl, M., et al., *[Time-allocation study of nurse and physician activities in the emergency department].* Med Klin Intensivmed Notfmed, 2020.
21. Ting, J.Y.S., *Emergency department presentations of patients with primary care complaints might engender negative staff attitudes that impact on quality of care.* Emergency Medicine Australasia, 2008. **20**(1): p. 91-92.
22. Basu, S., C. Yap, and S. Mason, *Examining the sources of occupational stress in an emergency department.* Occup Med (Lond), 2016. **66**(9): p. 737-742.
23. Xu, H.G., et al., *Effectiveness of interventions to reduce emergency department staff occupational stress and/or burnout: a systematic review.* JBI Evid Synth, 2020. **18**(6): p. 1156-1188.
24. Kansagra, S.M., et al., *A survey of workplace violence across 65 U.S. emergency departments.* Acad Emerg Med, 2008. **15**(12): p. 1268-74.
25. Carter, E.J., S.M. Pouch, and E.L. Larson, *Common infection control practices in the emergency department: a literature review.* Am J Infect Control, 2014. **42**(9): p. 957-62.
26. Liang, S.Y., et al., *Infection prevention in the emergency department.* Ann Emerg Med, 2014. **64**(3): p. 299-313. 6
27. Abraham, L.J., et al., *Morale, stress and coping strategies of staff working in the emergency department: A comparison of two different-sized departments.* Emerg Med Australas, 2018. **30**(3): p. 375-381.
28. Berlanda, S., et al., *Addressing Risks of Violence against Healthcare Staff in Emergency Departments: The Effects of Job Satisfaction and Attachment Style.* Biomed Res Int, 2019. **2019**: p. 5430870.
29. Baig, A., et al., *Correlation of serum cortisol levels and stress among medical doctors working in emergency departments.* J Coll Physicians Surg Pak, 2006. **16**(9): p. 576-80.

30. Komissarova, E.M. and M.A. Ermakova, *[Characteristics of arterial hypertension in psychoemotional burnout of emergency medical staffers].* Med Tr Prom Ekol, 2011(10): p. 19-23.
31. Kotov, A.V. and N.E. Revina, *Heart rate variability during "alarm stage" of burnout syndrome in emergency doctors.* Bull Exp Biol Med, 2012. **153**(5): p. 598-600.
32. Dutheil, F., et al., *Maximal tachycardia and high cardiac strain during night shifts of emergency physicians.* Int Arch Occup Environ Health, 2017. **90**(6): p. 467-480.
33. Rugless, M.J. and D.M. Taylor, *Sick leave in the emergency department: staff attitudes and the impact of job designation and psychosocial work conditions.* Emerg Med Australas, 2011. **23**(1): p. 39-45.
34. Lin, B.Y., et al., *Relationships of hospital-based emergency department culture to work satisfaction and intent to leave of emergency physicians and nurses.* Health Serv Manage Res, 2012. **25**(2): p. 68-77.
35. Stehman, C.R., et al., *Burnout, Drop Out, Suicide: Physician Loss in Emergency Medicine, Part I.* West J Emerg Med, 2019. **20**(3): p. 485-494.
36. Stehman, C.R., et al., *Erratum: This Article Corrects: "Burnout, Drop Out, Suicide: Physician Loss in Emergency Medicine, Part 1".* West J Emerg Med, 2019. **20**(5): p. 840-841.
37. Nicks, B.A. and D. Nelson, *Emergency department operations and management education in emergency medicine training.* World J Emerg Med, 2012. **3**(2): p. 98-101.
38. Elder, E.G., et al., *Work-based strategies/interventions to ameliorate stressors and foster coping for clinical staff working in emergency departments: a scoping review of the literature.* Australas Emerg Care, 2020.
39. Gulen, B., et al., *Serum S100B as a Surrogate Biomarker in the Diagnoses of Burnout and Depression in Emergency Medicine Residents.* Acad Emerg Med, 2016. **23**(7): p. 786-9.
40. Maas, M. and T. Güß, *[Patient safety -- mission for the future: The importance of Critical Incident Reporting Systems (CIRS) in clinical practice].* Anasthesiol Intensivmed Notfallmed Schmerzther, 2014. **49**(7-8): p. 466-72; quiz 473.
41. Beyer, M., et al., *[jeder-fehler-zaehlt.de: Content of and prospective benefits from a critical incident reporting and learning system (CIRS) for primary care].* Z Evid Fortbild Qual Gesundhwes, 2015. **109**(1): p. 62-8.
42. Babitsch, B., et al., *The relevance of cultural diversity on safety culture: a CIRS data analysis to identify problem areas and competency requirements of professionals in healthcare institutions.* GMS J Med Educ, 2020. **37**(2): p. Doc14.
43. Neuhaus, C., et al., *[Findings from 10 years of CIRS-AINS : An analysis of usepatterns and insights into new challenges].* Anaesthesist, 2020. **69**(11): p. 793-802.

44. Caponnetto, P., et al., *Quality of life, work motivation, burn-out and stress perceptions benefits of a stress management program by autogenic training for emergency room staff: A pilot study.* Ment Illn, 2018. **10**(2): p. 7913.
45. Dunne, P.J., et al., *Burnout in the emergency department: Randomized controlled trial of an attention-based training program.* J Integr Med, 2019. **17**(3): p. 173-180.
46. Schneider, A., M. Wehler, and M. Weigl, *Effects of work conditions on provider mental well-being and quality of care: a mixed-methods intervention study in the emergency department.* BMC Emerg Med, 2019. **19**(1): p. 1.
47. Morey, J.C., et al., *Error reduction and performance improvement in the emergency department through formal teamwork training: evaluation results of the MedTeams project.* Health Serv Res, 2002. **37**(6): p. 1553-81.
48. Goldman, E.F., et al., *Learning clinical versus leadership competencies in the emergency department: strategies, challenges, and supports of emergency medicine residents.* J Grad Med Educ, 2011. **3**(3): p. 320-5.
49. Jones, F., P. Podila, and C. Powers, *Creating a culture of safety in the emergency department: the value of teamwork training.* J Nurs Adm, 2013. **43**(4): p. 194-200.
50. Doby, V., *Leadership's role in eliminating workplace violence and changing perceptions in the emergency department.* J Emerg Nurs, 2015. **41**(1): p. 7. 7
51. Perry, S. J.*,* et al. *(2012). Human factors and ergonomics in the emergency department. Handbook of human factors and ergonomics in health care and patient safety. 2nd edn. Boca Raton, FL: Taylor & Francis Group, 709-22.*
52. Wears, R. L., & Perry, S. J. *(2002). Human factors and ergonomics in the emergency department.* An Emerg Med, 40(2), 206-212.

51. Husebø, S.E. and E. Olsen Ø, *Impact of clinical leadership in teams' course on quality, efficiency, responsiveness and trust in the emergency department: study protocol of a trailing research study.* BMJ Open, 2016. **6**(8): p. e011899.
52. Rixon, A., et al., *Communication and Influencing for ED Professionals: A training programme developed in the emergency department for the emergency department.* Emerg Med Australas, 2016. **28**(4): p. 404-11.
53. Aaronson, E.L., et al., *Training to Improve Communication Quality: An Efficient Interdisciplinary Experience for Emergency Department Clinicians.* Am J Med Qual, 2019. **34**(3): p. 260-265.
54. Lien, W.C., et al., *A leadership-based program can reduce boarding time of emergency department admissions.* Am J Emerg Med, 2019. **37**(4): p. 783-788.
55. Patterson, M.D., et al., *In situ simulation: detection of safety threats and teamwork training in a high risk emergency department.* BMJ Qual Saf, 2013. **22**(6): p. 468-77.
56. Patterson, M.D., et al., *Impact of multidisciplinary simulation-based training on patient safety in a paediatric emergency department.* BMJ Qual Saf, 2013. **22**(5): p. 383-93.
57. Sweeney, L.A., et al., *A simulation-based training program improves emergency department staff communication.* Am J Med Qual, 2014. **29**(2): p. 115-23.
58. Zun, L.S. and D. Moss, *Bonus/incentive programs to increase physician productivity in academic emergency medicine.* Am J Emerg Med, 1996. **14**(3): p. 334-6.
59. Etherington, J., et al., *Development, implementation and reliability assessment of an emergency physician performance evaluation tool.* Cjem, 2000. **2**(4): p. 237-45.
60. Magyar, J. and T. Theophilos, *Review article: debriefing critical incidents in the emergency department.* Emerg Med Australas, 2010. **22**(6): p. 499-506.
61. Kessler, D.O., A. Cheng, and P.C. Mullan, *Debriefing in the emergency department after clinical events: a practical guide.* Ann Emerg Med, 2015. **65**(6): p. 690-8.
62. Nadir, N.A., et al., *Characteristics of Real-Time, Non-Critical Incident Debriefing Practices in the Emergency Department.* West J Emerg Med, 2017. **18**(1): p. 146-151.
63. Cantu, L. and L. Thomas, *Baseline well-being, perceptions of critical incidents, and openness to debriefing in community hospital emergency department clinical staff before COVID-19, a cross-sectional study.* BMC Emerg Med, 2020. **20**(1): p. 82.
64. Coggins, A., et al., *Interdisciplinary clinical debriefing in the emergency department: an observational study of learning topics and outcomes.* BMC Emerg Med, 2020. **20**(1): p. 79.
65. Elder, E., A.N. Johnston, and J. Crilly, *Review article: systematic review of three key strategies designed to improve patient flow through the emergency department.* Emerg Med Australas, 2015. **27**(5): p. 394-404.

66. Chang, B.P. and K. Cato, *Tackling Burnout With Team Science: Nursing and Physician Collaborations on Improving Psychological Well-Being Among Emergency Clinicians.* Journal of Emergency Nursing, 2020. **46**(5): p. 557-559.
67. Howlett, M., et al., *Burnout in emergency department healthcare professionals is associated with coping style: a cross-sectional survey.* Emerg Med J, 2015. **32**(9): p. 722-7.
68. Wachs, P., et al., *Resilience skills as emergent phenomena: A study of emergency departments in Brazil and the United States.* Appl Ergon, 2016. **56**: p. 227-37.
69. Katari, R., *Regarding Wellness and Burnout Initiatives in Emergency Medicine.* Acad Emerg Med, 2018. **25**(5): p. 607-608.
70. Son, C., et al., *Resilient performance of emergency department: Patterns, models and strategies.* Safety Science, 2019. **120**: p. 362-373.
71. Watson, A.G., et al., *Self-reported modifying effects of resilience factors on perceptions of workload, patient outcomes, and burnout in physician-attendees of an international emergency medicine conference.* Psychol Health Med, 2019. **24**(10): p. 1220-1234.
72. Siddle, J., S. Tolleson-Rinehart, and J. Brice, *Survey of Emergency Department staff on disaster preparedness and training for Ebola virus disease.* Am J Disaster Med, 2016. **11**(1): p. 5-18.
73. Chen, S.C., K. Chang, and C.H. Kuo, *Emergency department infection control strategies in response to COVID-19.* Kaohsiung J Med Sci, 2020. **36**(7): p. 568-569.
74. Kung, C.T., et al., *Effective strategies to prevent in-hospital infection in the emergency department during the novel coronavirus disease 2019 pandemic.* J Microbiol Immunol Infect, 2020.
75. Schmitz, D., et al., *Association between personal protective equipment and SARS-CoV-2 infection risk in emergency department healthcare workers.* Eur J Emerg Med, 2020.
76. Walker, A.D., et al., *Development of an interactive curriculum and trainee-specific preparedness plan for emergency medicine residents.* Int J Emerg Med, 2020. **13**(1): p. 37. 8

77. Lang, E., et al., *Authentic emergency department leadership during a pandemic.* Cjem, 2020. **22**(4): p. 400-403.
78. Pothiawala, S., H.K. Lau, and A. Annathurai, *Regular versus extended shift outbreak roster in the emergency department and its impact on staff well-being.* Emerg Med J, 2020. **37**(8): p. 468.
79. de Wit, K., et al., *Canadian emergency physician psychological distress and burnout during the first 10 weeks of COVID-19: A mixed-methods study.* J Am Coll Emerg Physicians Open, 2020.
80. Dorsett, M., *Point of no return: COVID-19 and the U.S. healthcare system: An emergency physician's perspective.* Sci Adv, 2020. **6**(26): p. eabc5354.
81. Rodriguez, R.M., et al., *Academic Emergency Medicine Physicians' Anxiety Levels, Stressors, and Potential Stress Mitigation Measures During the Acceleration Phase of the COVID-19 Pandemic.* Acad Emerg Med, 2020.
