# Supplementary material for: Key factors for sustainable working conditions in emergency departments: an EUSEM-initiated, Europe-wide consensus survey
Source: Eur J Emerg Med. 2024 Jul 19;32(1):29–37. doi: 10.1097/MEJ.0000000000001159 (PMC11665970; doi:10.1097/MEJ.0000000000001159)
Supplement: Supplementary file 2 [file ejem-32-29-s002.pdf]

Appendix A-3

List of Search Terms

1) delphi[Title/Abstract] AND emergency[Title/Abstract]
2) interview[Title/Abstract] AND emergency[Title/Abstract]
3) work conditions[Title/Abstract] AND emergency[Title/Abstract]
4) work stress[Title/Abstract] AND emergency[Title/Abstract]
5) Job stress[Title/Abstract] AND emergency[Title/Abstract]
6) Job strain[Title/Abstract] AND emergency[Title/Abstract]
7) work characteristics[Title/Abstract] AND emergency[Title/Abstract]
8) employee strain[Title/Abstract] AND emergency[Title/Abstract]
9) employee[Title/Abstract] AND emergency[Title/Abstract]
10) psychosocial work factors[Title/Abstract] AND emergency[Title/Abstract]
11) provider mental well-being[Title/Abstract] AND emergency[Title/Abstract]
12) work factors[Title/Abstract] AND emergency[Title/Abstract]
13) psychosocial[Title/Abstract] AND emergency[Title/Abstract]
14) provider[Title/Abstract] AND emergency[Title/Abstract]
15) providers[Title/Abstract] AND emergency[Title/Abstract]
16) conditions[Title/Abstract] AND emergency[Title/Abstract]
17) well-being[Title/Abstract] AND emergency[Title/Abstract]
18) stress[Title/Abstract] AND emergency[Title/Abstract]
19) strain[Title/Abstract] AND emergency[Title/Abstract]
20) quality of care[Title/Abstract] AND emergency[Title/Abstract]
21) work-related strain[Title/Abstract] AND emergency[Title/Abstract]
22) job demands[Title/Abstract] AND emergency[Title/Abstract]
23) demands[Title/Abstract] AND emergency[Title/Abstract]
24) burnout[Title/Abstract] AND emergency[Title/Abstract]
25) job satisfaction[Title/Abstract] AND emergency[Title/Abstract]
26) work satisfaction[Title/Abstract] AND emergency[Title/Abstract]
27) satisfaction[Title/Abstract] AND emergency[Title/Abstract]
28) physician[Title/Abstract] AND emergency[Title/Abstract]
29) Job condition AND emergency[Title/Abstract]
30) Job characteristic AND emergency[Title/Abstract]
31) Job factors AND emergency[Title/Abstract]
32) Work-related AND emergency[Title/Abstract]
33) Work AND emergency[Title/Abstract]
34) Work stressors AND emergency[Title/Abstract]
35) Work fatigue AND emergency[Title/Abstract]
36) Job-related AND emergency[Title/Abstract]
37) Job AND emergency[Title/Abstract]
38) Wellness AND emergency[Title/Abstract]
39) Workplace AND emergency[Title/Abstract]
40) Health AND emergency[Title/Abstract]
41) Environment AND emergency[Title/Abstract]
42) Quality of life AND emergency[Title/Abstract]
43) Occupation AND emergency[Title/Abstract]
44) Mental stress AND emergency[Title/Abstract]
45) Emotional distress AND emergency[Title/Abstract]
