# Supplementary material for: Key factors for sustainable working conditions in emergency departments: an EUSEM-initiated, Europe-wide consensus survey
Source: Eur J Emerg Med. 2024 Jul 19;32(1):29–37. doi: 10.1097/MEJ.0000000000001159 (PMC11665970; doi:10.1097/MEJ.0000000000001159)
Supplement: Supplementary file 3 [file ejem-32-29-s003.pdf]

EUSEM cross-European Emergency department (ED) expert-based interview guideline (Delphi method round 1) [1]

Dear Sir or Madam,

Thank you very much for your interest in our expert survey and your consent to participate.

In order to gain preliminary insights into the work situation of ED physicians in your country and hospital, it is important to obtain experts' statements.

We prepared a list of questions that address several aspects of ED physicians' work life, well-being at the job, and self-perceived quality and safety of patient care. Furthermore, we are interested in your knowledge and appraisal of intervention approaches on previously mentioned aspects and of potential changes due to the COVID-19 pandemic.

We ask you to report us your individual opinions on the following issues:

(1) ED physicians' work life, working conditions and work-related key factors

a. For your Emergency Department, please briefly state **work-related factors** of physicians' work life.

b. Please give a concise insight into the following **work life aspects** of your ED work:

Infrastructure & organization

- Clinic scope (e.g., category; specialties; ward beds; ICU & IMC beds; ED staff; annual out- & in-patient volume) [2]
- Triage system (e.g., number & definition of categories) [3, 4]
- ED physical work environment (e.g., workstation layout; space; privacy; lightning; noise; vibration; temperature; humidity; air quality) [5]
- (Un-)beneficially influential societal, economic, ecological & political factors [5]

Personnel & employment conditions

- Work system (e.g., schedules & shifts; rotation into inpatient wards & ICU/IMC; necessity of performing research & teaching) [5-7]
- Skills and qualification: ratio & definition (e.g., seniors vs juniors; physicians vs nurses; skill mix, gender & cultural diversity ratio; classification of different experience levels) [2, 5, 8]
- Staffing levels & determination (e.g., calculation approaches; *mean physician time per patient* vs *workplace method*; minimal staffing levels) [2, 8-10]
- Types of contracts (e.g., ratio of part- vs full-time; unlimited vs limited; permanent vs temporary rotation into the ED; average annual full-time salaries & on-call payments; additional compensation) [4, 7, 11]
- Overtime & compensation (e.g., average amount of monthly overtime; time off in lieu vs additional payment; electronical check-in/-out vs manual time diaries) [4, 7, 11]

(Information) Tools & technology

- Health information technologies / health records (e.g., documentation: average time per patient, computerized vs paper-based records & provider order entries; hand-held systems; patchwork of software tools; translation tools) [5]
- Availability of diagnostic & intervention technologies
- Introduction, skills training & free resource access [5]
- Maintenance & cleaning (e.g., real-time action in case of malfunction & deficits) [5]

Intra- & inter-professional collaboration

- Department responsibility for: (a) triage, (b) stabilization/resuscitation, (c) bed management, (d) diagnostic procedure, (e) immediate therapy, (f) disposition? [4]

(2) Influences (2a) and outcomes (2b) of ED work on ED physicians' well-being on the job and self-perceived quality and safety of patient care

a. For your Emergency Department, please briefly state **influences of ED work** on physicians' well-being on the job and on the self-perceived quality and safety of patient care (2a).

b. Please offer a concise insight into the following **influences** of your ED work by (a) putting them in a personal order from relevance to irrelevance and (b) adding unconsidered factors (2a):

Organizational influences

- **Job satisfaction:**
  ❶ Job intellectuality/content ❷ Task significance ❸ Work challenge, variation & interdisciplinary interaction ❹ Job autonomy ❺ Job control & participation in decision-making ❻ Work-life balance ❼ Job security ❽ Prospects & promotion ❾ Salary/payment ❿ Social esteem & respectability [2, 5, 11, 12]

- **Job dissatisfaction:**
  ❶ Chronic cognitive workload ❷ Time pressure & lack of breaks ❸ Workflow interruptions & multitasking ❹ Shift work ❺ Employee turnover, fluctuation, shortages & understaffing ❻ Overcrowding ❼ In-/outflow deficits & lack of patient information (transfer) ❽ Limited trainee authority, autonomy & job control ❾ Administrative & coordinative duties (e.g., interpreters/translators; bed management) ❿ Work-life imbalance ⓫ Delay in triage, diagnostic & therapy ⓬ Profitability ⓭ (Information) technology & internal/external environment deficits ⓮ Insufficient medication, resource & isolation supply [2, 4, 5, 7, 11, 13-20]

Provider influences

- **Job satisfaction:**
  ① Resilience & coping strategies ② Personal strong work ethic & motivation ③ Work experience & utilization of skills ④ Teamwork & social climate ⑤ Peer credibility & reputation ⑥ Positive co-workers' relationships ⑦ Conscientiousness & adequate risk-taking ⑧ Persistent presence of senior doctors, positive leader behaviour, sufficient supervisor support & teaching ⑨ Strong communication & feedback culture ⑩ Flat hierarchies [2, 4, 5, 11-13, 21-23]

- **Job dissatisfaction:**
  ① Lack of resilience & coping mechanisms ② Negative leader behaviour, lack of supervisor support & teaching ③ Problematic co-workers' relationships ④ Tense atmosphere ⑤ Low presence of senior doctors ⑥ Lack of communication & feedback culture ⑦ Steep hierarchies ⑧ Non-compliance with guidelines & algorithms ⑨ Under-/overconscientiousness ⑩ Inadequate risk-taking/averseness ⑪ Medical errors & adverse effects ⑫ Task overlap & interaction deficits (e.g., physicians vs nursing; ED vs other specialties; resuscitation room) ⑬ Lack of organizational skills ⑭ Lack of introduction into new procedures & structures [2, 4, 5, 11-13, 21-23]

Patient influences

- **Job satisfaction:**
  ❶ Grateful feedback ❷ Patient case complexity ❸ Participation in patients' private life

- **Job dissatisfaction:**
  ❶ Primary care complaints ❷ Patient case complexity ❸ Family & social concerns ❹ Non-compliance ❺ Breaking bad news & death ❻ Excessive claims & self-diagnostics ❼ Ungrateful feedback ❽ Verbal & physical violence ❾ Litigations [2, 4, 5, 7, 11, 14, 21]

c. For your Emergency Department, please briefly state **outcomes of negative ED work** on physicians' well-being on the job and on the self-perceived quality and safety of patient care (2b).

d. Please provide a concise insight into the following **outcomes** of your ED work (2b):

- *Outcomes on* ***physical health*** (e.g., musculoskeletal pain; (needlestick) injuries; infections; radiation, fatigue) [12, 24-28]

- *Outcomes on* ***mental health*** (e.g., internal tension; anxiety; sadness; despair; compassion fatigue; exhaustion; burnout; depression; isolation; PTSD; psychoses; personality disorders) [2, 5, 11, 12, 23]

- *Outcomes on* ***psychosomatic complaints*** (e.g., hypertension; tachycardia; sleep disturbances; skin problems; gastroesophageal reflux; diabetes; eating disorders) [29-32]

- *Outcomes on* ***behavioural patterns*** (e.g., substance abuse; intentions to leave; drop-outs; sick leaves; self-harm; suicidal ideation & rates) [2, 5, 33-36]

- *Outcomes on the* ***self-perceived quality & safety of patient care*** (e.g., feedback loop: patient -> management -> physician -> patient; challenges of patient handovers)

(3) Intervention approaches to improve ED physicians' work life, well-being and self-perceived quality and safety of patient care (3a); success/failure and effectiveness/ineffectiveness (3b)

a. For your Emergency Department, please briefly state **intervention strategies** to improve physicians' work life, well-being on the job and self-perceived quality & safety of patient care (3a).

b. Please give a concise insight into the following **intervention approaches** of your ED work and add unconsidered existing intervention approaches (3a):

- ***Interventions on organizational level***

  - ED expansion & modernization (e.g., improvement of internal environment concepts & information technology; non-health professionals for call centers & secretaries)
  - Standardized & evidence-based training concepts (e.g., Emergency care as autonomous specialty with official job descriptions) [37, 38]
  - Roster redesign (e.g., throughout collective agreements by trade unions; compressed work weeks & less on-calls) [37, 38]
  - Regular health checks & mental health interventions (e.g., assistance/consultation by mental health experts & occupational physicians / company medical officers) [30, 39]
  - Measurement instruments & feedback platforms (e.g., employee surveys; CIRS) [40-46]
  - Networking opportunities (e.g., platforms for exchange of experiences, opinions & criticism) [6]

- ***Interventions on team level***

  - Skill training (e.g., (simulation-based) resuscitation room; team building; communication; violence management; leadership) [38, 47-57]
  - Feedback & performance evaluations (e.g., junior doctors' representatives; quality circles; counselling; debriefings; mentor talks; peer reviews; incentive systems) [5, 38, 58-64]
  - Collaborations between nursing & physician staff (e.g., physician-assisted triage; medical assessment units; nurse practitioners & clinical initiative nurses) [65, 66]

- ***Interventions on individual level***

  - Physical fitness programs (e.g., lifestyle interventions; physiotherapy; pharmacological & herbal interventions) [12, 23, 38, 67-71]
  - Mental fitness programs (e.g., resilience & coping mechanism training; task-oriented vs emotion-oriented; mindfulness & relaxation techniques; hypnosis) [12, 23, 38, 67-71]

c. According to you, (why) are the already implemented interventions within your ED likely to be successful or non-successful (3b)?

d. What kind of **additional intervention approaches** would you personally deem effective to address ED physicians' working conditions, well-being on the job and self-perceived quality and safety of patient care (3b)?

(4) ED physicians' work life, working conditions, work-related key factors, well-being, self-perceived quality and safety of patient care and intervention approaches during the COVID-19 pandemic

a. For your Emergency Department, please briefly state **changes and adjustments** for physicians caused **by the COVID-19 pandemic**.

b. Please give a concise insight into the following **COVID-19 pandemic aspects** of your ED work:

- *What **major precautions** have been taken **to prevent exposure** to infection hazards (a) pre-COVID-19 and (b) specifically due to COVID-19* (e.g., quarantining; PPE; testing; vaccinations)*? Which prevention aspects have not been considered enough yet?* [5, 72-76]

- ***Work system** changes* (e.g., separate ED units; excessive workload; short-time allowance; rerostering & reallocation of staff for compensation of staff shortages & sick leaves; shortage in resources; leadership behaviour & communication strategies) [77, 78]

- *Impacts on **physical health*** (e.g., infections; violence; PPE overheating; skin & respiratory irritations; Long-COVID-syndrome) [79-81]

- *Impacts on **mental health*** (e.g., anxiety of infections; neglect of individual coping & resilience mechanisms; increased confrontation with patients' panic attacks; social isolation & domestic violence; aggravated work-life imbalance; exhaustion; burnout; depression) [79-81]

- *Impacts on **self-perceived quality and safety of patient care*** (e.g., reduced direct physical diagnostics & treatment; CT scanning overuse; misdiagnoses & misperceptions; aggravated untreated preconditions)

- *Impacts on **already implemented or planned intervention approaches*** to improve ED physicians' work life, well-being and self-perceived quality and safety of patient care

(5) Finally, a few questions concerning your professional background:

- What is your highest professional and academic qualification?

- What is your current role in emergency medicine?

- Please, rate your own impact & influence on emergency medicine as an ED expert?

- According to you, does your opinion only represent your specific local or also the generalized nationwide emergency care situation?

Thank you very much for your time and responses.

We very much appreciate your participation.

References

1. Iqbal, S. and L. Pipon-Young, *The Delphi method.* Psychologist, 2009. **22**: p. 598-601.
2. Schneider, A. and M. Weigl, *Associations between psychosocial work factors and provider mental well-being in emergency departments: A systematic review.* PLoS One, 2018. **13**(6): p. e0197375.
3. Sakr, M. and J. Wardrope, *Casualty, accident and emergency, or emergency medicine, the evolution.* J Accid Emerg Med, 2000. **17**(5): p. 314-9.
4. Exadaktylos, A.K. and H. Zimmermann, *[Interdisciplinary emergency response units: he who comes too late is punished by life and or by the hospital management!].* Dtsch Med Wochenschr, 2009. **134**(23): p. 1236-7.
5. Carayon, P., et al., *Work system design for patient safety: the SEIPS model.* Qual Saf Health Care, 2006. **15 Suppl 1**: p. i50-8.
6. Arora, M., et al., *Review article: burnout in emergency medicine physicians.* Emerg Med Australas, 2013. **25**(6): p. 491-5.
7. Johnston, A., et al., *Review article: Staff perception of the emergency department working environment: Integrative review of the literature.* Emerg Med Australas, 2016. **28**(1): p. 7-26.
8. Weigl, M., et al., *Work stress, burnout, and perceived quality of care: a cross-sectional study among hospital pediatricians.* Eur J Pediatr, 2015. **174**(9): p. 1237-46.
9. Behringer, W. and C. Dodt, *[Physician staffing and shift work schedules : Concepts for emergency and intensive care medicine].* Med Klin Intensivmed Notfmed, 2020. **115**(6): p. 449-457.
10. Wrede, J., H. Wrede, and W. Behringer, *Emergency Department Mean Physician Time per Patient and Workload Predictors ED-MPTPP.* J Clin Med, 2020. **9**(11).
11. Weigl, M. and A. Schneider, *Associations of work characteristics, employee strain and self-perceived quality of care in Emergency Departments: A cross-sectional study.* Int Emerg Nurs, 2017. **30**: p. 20-24.
12. Boutou, A., et al., *Burnout syndrome among emergency medicine physicians: an update on its prevalence and risk factors.* Eur Rev Med Pharmacol Sci, 2019. **23**(20): p. 9058-9065.
13. Holden, R.J., et al., *SEIPS 2.0: a human factors framework for studying and improving the work of healthcare professionals and patients.* Ergonomics, 2013. **56**(11): p. 1669-86.
14. Carayon, P., et al., *Human factors systems approach to healthcare quality and patient safety.* Appl Ergon, 2014. **45**(1): p. 14-25.
15. Carayon, P., et al., *SEIPS 3.0: Human-centered design of the patient journey for patient safety.* Appl Ergon, 2020. **84**: p. 103033.
16. Weigl, M., et al., *Workflow interruptions and stress atwork: a mixed-methods study among physicians and nurses of a multidisciplinary emergency department.* BMJ Open, 2017. **7**(12): p. e019074.
17. Schneider, A., et al., *Physicians' and nurses' work time allocation and workflow interruptions in emergency departments: a comparative time-motion study across two countries.* Emerg Med J, 2020.
18. Weigl, M., et al., *Workflow disruptions and provider situation awareness in acute care: An observational study with emergency department physicians and nurses.* Appl Ergon, 2020. **88**: p. 103155.
19. Schneider, A., M. Wehler, and M. Weigl, *Provider interruptions and patient perceptions of care: an observational study in the emergency department.* BMJ Qual Saf, 2019. **28**(4): p. 296-304.
20. Weigl, M., et al., *[Time-allocation study of nurse and physician activities in the emergency department].* Med Klin Intensivmed Notfmed, 2020.
21. Ting, J.Y.S., *Emergency department presentations of patients with primary care complaints might engender negative staff attitudes that impact on quality of care.* Emergency Medicine Australasia, 2008. **20**(1): p. 91-92.
22. Basu, S., C. Yap, and S. Mason, *Examining the sources of occupational stress in an emergency department.* Occup Med (Lond), 2016. **66**(9): p. 737-742.
23. Xu, H.G., et al., *Effectiveness of interventions to reduce emergency department staff occupational stress and/or burnout: a systematic review.* JBI Evid Synth, 2020. **18**(6): p. 1156-1188.
24. Kansagra, S.M., et al., *A survey of workplace violence across 65 U.S. emergency departments.* Acad Emerg Med, 2008. **15**(12): p. 1268-74.
25. Carter, E.J., S.M. Pouch, and E.L. Larson, *Common infection control practices in the emergency department: a literature review.* Am J Infect Control, 2014. **42**(9): p. 957-62.
26. Liang, S.Y., et al., *Infection prevention in the emergency department.* Ann Emerg Med, 2014. **64**(3): p. 299-313.

27. Abraham, L.J., et al., *Morale, stress and coping strategies of staff working in the emergency department: A comparison of two different-sized departments.* Emerg Med Australas, 2018. **30**(3): p. 375-381.
28. Berlanda, S., et al., *Addressing Risks of Violence against Healthcare Staff in Emergency Departments: The Effects of Job Satisfaction and Attachment Style.* Biomed Res Int, 2019. **2019**: p. 5430870.
29. Baig, A., et al., *Correlation of serum cortisol levels and stress among medical doctors working in emergency departments.* J Coll Physicians Surg Pak, 2006. **16**(9): p. 576-80.
30. Komissarova, E.M. and M.A. Ermakova, *[Characteristics of arterial hypertension in psychoemotional burnout of emergency medical staffers].* Med Tr Prom Ekol, 2011(10): p. 19-23.
31. Kotov, A.V. and N.E. Revina, *Heart rate variability during "alarm stage" of burnout syndrome in emergency doctors.* Bull Exp Biol Med, 2012. **153**(5): p. 598-600.
32. Dutheil, F., et al., *Maximal tachycardia and high cardiac strain during night shifts of emergency physicians.* Int Arch Occup Environ Health, 2017. **90**(6): p. 467-480.
33. Rugless, M.J. and D.M. Taylor, *Sick leave in the emergency department: staff attitudes and the impact of job designation and psychosocial work conditions.* Emerg Med Australas, 2011. **23**(1): p. 39-45.
34. Lin, B.Y., et al., *Relationships of hospital-based emergency department culture to work satisfaction and intent to leave of emergency physicians and nurses.* Health Serv Manage Res, 2012. **25**(2): p. 68-77.
35. Stehman, C.R., et al., *Burnout, Drop Out, Suicide: Physician Loss in Emergency Medicine, Part I.* West J Emerg Med, 2019. **20**(3): p. 485-494.
36. Stehman, C.R., et al., *Erratum: This Article Corrects: "Burnout, Drop Out, Suicide: Physician Loss in Emergency Medicine, Part 1".* West J Emerg Med, 2019. **20**(5): p. 840-841.
37. Nicks, B.A. and D. Nelson, *Emergency department operations and management education in emergency medicine training.* World J Emerg Med, 2012. **3**(2): p. 98-101.
38. Elder, E.G., et al., *Work-based strategies/interventions to ameliorate stressors and foster coping for clinical staff working in emergency departments: a scoping review of the literature.* Australas Emerg Care, 2020.
39. Gulen, B., et al., *Serum S100B as a Surrogate Biomarker in the Diagnoses of Burnout and Depression in Emergency Medicine Residents.* Acad Emerg Med, 2016. **23**(7): p. 786-9.
40. Maas, M. and T. Güß, *[Patient safety -- mission for the future: The importance of Critical Incident Reporting Systems (CIRS) in clinical practice].* Anasthesiol Intensivmed Notfallmed Schmerzther, 2014. **49**(7-8): p. 466-72; quiz 473.
41. Beyer, M., et al., *[jeder-fehler-zaehlt.de: Content of and prospective benefits from a critical incident reporting and learning system (CIRS) for primary care].* Z Evid Fortbild Qual Gesundhwes, 2015. **109**(1): p. 62-8.
42. Babitsch, B., et al., *The relevance of cultural diversity on safety culture: a CIRS data analysis to identify problem areas and competency requirements of professionals in healthcare institutions.* GMS J Med Educ, 2020. **37**(2): p. Doc14.
43. Neuhaus, C., et al., *[Findings from 10 years of CIRS-AINS : An analysis of usepatterns and insights into new challenges].* Anaesthesist, 2020. **69**(11): p. 793-802.
44. Caponnetto, P., et al., *Quality of life, work motivation, burn-out and stress perceptions benefits of a stress management program by autogenic training for emergency room staff: A pilot study.* Ment Illn, 2018. **10**(2): p. 7913.
45. Dunne, P.J., et al., *Burnout in the emergency department: Randomized controlled trial of an attention-based training program.* J Integr Med, 2019. **17**(3): p. 173-180.
46. Schneider, A., M. Wehler, and M. Weigl, *Effects of work conditions on provider mental well-being and quality of care: a mixed-methods intervention study in the emergency department.* BMC Emerg Med, 2019. **19**(1): p. 1.
47. Morey, J.C., et al., *Error reduction and performance improvement in the emergency department through formal teamwork training: evaluation results of the MedTeams project.* Health Serv Res, 2002. **37**(6): p. 1553-81.
48. Goldman, E.F., et al., *Learning clinical versus leadership competencies in the emergency department: strategies, challenges, and supports of emergency medicine residents.* J Grad Med Educ, 2011. **3**(3): p. 320-5.
49. Jones, F., P. Podila, and C. Powers, *Creating a culture of safety in the emergency department: the value of teamwork training.* J Nurs Adm, 2013. **43**(4): p. 194-200.
50. Doby, V., *Leadership's role in eliminating workplace violence and changing perceptions in the emergency department.* J Emerg Nurs, 2015. **41**(1): p. 7.

51. Husebø, S.E. and E. Olsen Ø, *Impact of clinical leadership in teams' course on quality, efficiency, responsiveness and trust in the emergency department: study protocol of a trailing research study.* BMJ Open, 2016. **6**(8): p. e011899.
52. Rixon, A., et al., *Communication and Influencing for ED Professionals: A training programme developed in the emergency department for the emergency department.* Emerg Med Australas, 2016. **28**(4): p. 404-11.
53. Aaronson, E.L., et al., *Training to Improve Communication Quality: An Efficient Interdisciplinary Experience for Emergency Department Clinicians.* Am J Med Qual, 2019. **34**(3): p. 260-265.
54. Lien, W.C., et al., *A leadership-based program can reduce boarding time of emergency department admissions.* Am J Emerg Med, 2019. **37**(4): p. 783-788.
55. Patterson, M.D., et al., *In situ simulation: detection of safety threats and teamwork training in a high risk emergency department.* BMJ Qual Saf, 2013. **22**(6): p. 468-77.
56. Patterson, M.D., et al., *Impact of multidisciplinary simulation-based training on patient safety in a paediatric emergency department.* BMJ Qual Saf, 2013. **22**(5): p. 383-93.
57. Sweeney, L.A., et al., *A simulation-based training program improves emergency department staff communication.* Am J Med Qual, 2014. **29**(2): p. 115-23.
58. Zun, L.S. and D. Moss, *Bonus/incentive programs to increase physician productivity in academic emergency medicine.* Am J Emerg Med, 1996. **14**(3): p. 334-6.
59. Etherington, J., et al., *Development, implementation and reliability assessment of an emergency physician performance evaluation tool.* Cjem, 2000. **2**(4): p. 237-45.
60. Magyar, J. and T. Theophilos, *Review article: debriefing critical incidents in the emergency department.* Emerg Med Australas, 2010. **22**(6): p. 499-506.
61. Kessler, D.O., A. Cheng, and P.C. Mullan, *Debriefing in the emergency department after clinical events: a practical guide.* Ann Emerg Med, 2015. **65**(6): p. 690-8.
62. Nadir, N.A., et al., *Characteristics of Real-Time, Non-Critical Incident Debriefing Practices in the Emergency Department.* West J Emerg Med, 2017. **18**(1): p. 146-151.
63. Cantu, L. and L. Thomas, *Baseline well-being, perceptions of critical incidents, and openness to debriefing in community hospital emergency department clinical staff before COVID-19, a cross-sectional study.* BMC Emerg Med, 2020. **20**(1): p. 82.
64. Coggins, A., et al., *Interdisciplinary clinical debriefing in the emergency department: an observational study of learning topics and outcomes.* BMC Emerg Med, 2020. **20**(1): p. 79.
65. Elder, E., A.N. Johnston, and J. Crilly, *Review article: systematic review of three key strategies designed to improve patient flow through the emergency department.* Emerg Med Australas, 2015. **27**(5): p. 394-404.
66. Chang, B.P. and K. Cato, *Tackling Burnout With Team Science: Nursing and Physician Collaborations on Improving Psychological Well-Being Among Emergency Clinicians.* Journal of Emergency Nursing, 2020. **46**(5): p. 557-559.
67. Howlett, M., et al., *Burnout in emergency department healthcare professionals is associated with coping style: a cross-sectional survey.* Emerg Med J, 2015. **32**(9): p. 722-7.
68. Wachs, P., et al., *Resilience skills as emergent phenomena: A study of emergency departments in Brazil and the United States.* Appl Ergon, 2016. **56**: p. 227-37.
69. Katari, R., *Regarding Wellness and Burnout Initiatives in Emergency Medicine.* Acad Emerg Med, 2018. **25**(5): p. 607-608.
70. Son, C., et al., *Resilient performance of emergency department: Patterns, models and strategies.* Safety Science, 2019. **120**: p. 362-373.
71. Watson, A.G., et al., *Self-reported modifying effects of resilience factors on perceptions of workload, patient outcomes, and burnout in physician-attendees of an international emergency medicine conference.* Psychol Health Med, 2019. **24**(10): p. 1220-1234.
72. Siddle, J., S. Tolleson-Rinehart, and J. Brice, *Survey of Emergency Department staff on disaster preparedness and training for Ebola virus disease.* Am J Disaster Med, 2016. **11**(1): p. 5-18.
73. Chen, S.C., K. Chang, and C.H. Kuo, *Emergency department infection control strategies in response to COVID-19.* Kaohsiung J Med Sci, 2020. **36**(7): p. 568-569.
74. Kung, C.T., et al., *Effective strategies to prevent in-hospital infection in the emergency department during the novel coronavirus disease 2019 pandemic.* J Microbiol Immunol Infect, 2020.
75. Schmitz, D., et al., *Association between personal protective equipment and SARS-CoV-2 infection risk in emergency department healthcare workers.* Eur J Emerg Med, 2020.
76. Walker, A.D., et al., *Development of an interactive curriculum and trainee-specific preparedness plan for emergency medicine residents.* Int J Emerg Med, 2020. **13**(1): p. 37.

77. Lang, E., et al., *Authentic emergency department leadership during a pandemic.* Cjem, 2020. **22**(4): p. 400-403.
78. Pothiawala, S., H.K. Lau, and A. Annathurai, *Regular versus extended shift outbreak roster in the emergency department and its impact on staff well-being.* Emerg Med J, 2020. **37**(8): p. 468.
79. de Wit, K., et al., *Canadian emergency physician psychological distress and burnout during the first 10 weeks of COVID-19: A mixed-methods study.* J Am Coll Emerg Physicians Open, 2020.
80. Dorsett, M., *Point of no return: COVID-19 and the U.S. healthcare system: An emergency physician's perspective.* Sci Adv, 2020. **6**(26): p. eabc5354.
81. Rodriguez, R.M., et al., *Academic Emergency Medicine Physicians' Anxiety Levels, Stressors, and Potential Stress Mitigation Measures During the Acceleration Phase of the COVID-19 Pandemic.* Acad Emerg Med, 2020.
